# The specificity of Pavlovian regulation is associated with recovery from depression

**DOI:** 10.1017/S0033291715002597

**Published:** 2016-04

**Authors:** Q. J. M. Huys, M. Gölzer, E. Friedel, A. Heinz, R. Cools, P. Dayan, R. J. Dolan

**Affiliations:** 1 Wellcome Trust Centre for Neuroimaging, University College London, London, UK; 2 Gatsby Computational Neuroscience Unit, University College London, London, UK; 3 Translational Neuromodeling Unit, Institute for Biomedical Engineering, University of Zürich and Swiss Federal Institute of Technology (ETH), Zürich, Switzerland; 4 Centre for Addictive Disorders, Department of Psychiatry, Psychotherapy and Psychosomatics, Hospital of Psychiatry Zürich, University of Zürich, Zürich, Switzerland; 5 Charité Universitätsmedizin Berlin, Campus Charité Mitte, Berlin, Germany; 6 Radboud University Medical Center, Donders Institute for Brain, Cognition and Behaviour, Centre for Cognitive Neuroimaging, Nijmegen, The Netherlands; 7 Max Planck UCL Centre for Computational Psychiatry and Ageing Research, London, UK; 8 Berlin School of Mind and Brain, Humboldt-Universität zu Berlin, Berlin, Germany

**Keywords:** Decision-making, emotional reactions, major depressive disorder, Pavlovian-instrumental transfer

## Abstract

**Background:**

Changes in reflexive emotional responses are hallmarks of depression, but how emotional reflexes make an impact on adaptive decision-making in depression has not been examined formally. Using a Pavlovian-instrumental transfer (PIT) task, we compared the influence of affectively valenced stimuli on decision-making in depression and generalized anxiety disorder compared with healthy controls; and related this to the longitudinal course of the illness.

**Method:**

A total of 40 subjects with a current DSM-IV-TR diagnosis of major depressive disorder, dysthymia, generalized anxiety disorder, or a combination thereof, and 40 matched healthy controls performed a PIT task that assesses how instrumental approach and withdrawal behaviours are influenced by appetitive and aversive Pavlovian conditioned stimuli (CSs). Patients were followed up after 4–6 months. Analyses focused on patients with depression alone (*n* = 25).

**Results:**

In healthy controls, Pavlovian CSs exerted action-specific effects, with appetitive CSs boosting active approach and aversive CSs active withdrawal. This action-specificity was absent in currently depressed subjects. Greater action-specificity in patients was associated with better recovery over the follow-up period.

**Conclusions:**

Depression is associated with an abnormal influence of emotional reactions on decision-making in a way that may predict recovery.

## Introduction

Computational theories of valuation provide a quantitative framework linking emotions to choices. They have carved out distinguishable but interacting decision-making systems (Daw & Dayan, 2014) including a reflective goal-directed system where choices are flexibly based on a prospective consideration of likely outcomes, and an ‘automatic’ Pavlovian system that inflexibly mandates evolutionarily ingrained reflex responses to emotional stimuli (Dayan *et al.* 2006; Guitart-Masip *et al.* 2011). In the context of depression, the interaction between these systems is particularly worth investigating as it might provide a quantitative handle on how reflexive emotional responses shape reflective cognitions, and vice versa (Huys *et al.* 2015).

Depression appears to alter reflexive emotional responses. In the appetitive domain, depression reduces reflexively evoked approach responses to a variety of appetitive stimuli such as films, images or monetary incentives (Rottenberg, 2005; Steele *et al.* 2007; Bylsma *et al.* 2008; Eshel & Roiser, 2010). If approach to positive situations or stimuli no longer occurs reflexively, but only after effortful reflection, then this might reduce the perceived ease of earning rewards and their experienced prevalence, both aspects of anhedonia. On the aversive side, an impairment in Pavlovian forms of behavioural inhibition (possibly mediated by serotonin: Dayan & Huys, 2008; Crockett *et al.* 2009; Geurts *et al.* 2013*a*) could suppress an automatic avoidance of potentially stressful situations and help explain why depression seems to both cause and be caused by stressful life events (Kendler *et al.* 1999, 2000).

Importantly, failures in reflexive Pavlovian approach or avoidance might have consequences for the internal working of other aspects of emotion (Huys *et al.* 2012): Because internal choices about what to think about are in many ways similar to external actions about what to do (Anderson & Oates, 2007; Huys *et al.* 2015), failures in internally directed Pavlovian approach might reduce positive emotion regulation strategies, such as the tendency to imagine positive events to up-regulate one's mood (Joormann & Vanderlind, 2014). A failure to consider positive explanations of events reflexively might contribute to negative cognitive distortions. Conversely, a reflexive sense of overwhelming failure might prevent reflective problem-solving (Elliott *et al.* 1997).

How reflexive Pavlovian responses interact with, and influence, other decision-making processes has yet to be studied explicitly in the setting of depression. To address this lacuna, we employed a Pavlovian-instrumental transfer (PIT) task and studied the influence of reflexive responses to incidental valued stimuli on ongoing deliberations about whether to approach and withdraw.

**Fig. 1.**
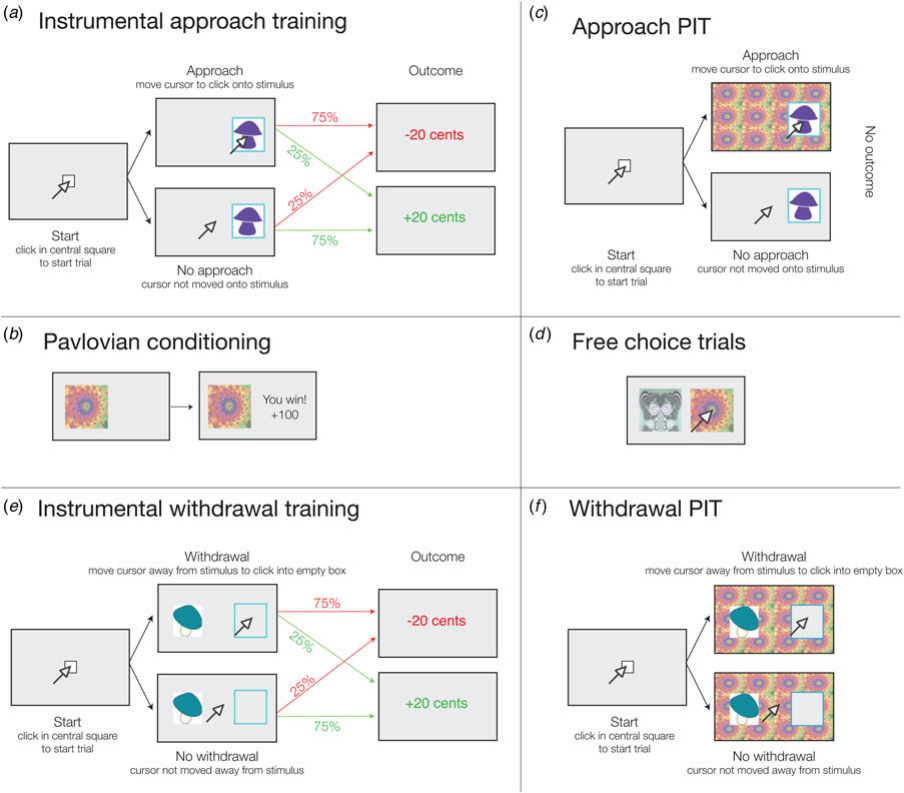
Task description. The task consisted of counterbalanced approach and withdrawal blocks, each subdivided into three parts: instrumental training, Pavlovian conditioning and Pavlovian-instrumental transfer (PIT). (*a*) Instrumental approach training. Subjects started each trial by clicking inside a central square. Subjects were told they were collecting mushrooms in the woods and had to choose whether to move the cursor towards the mushroom (instrumental stimulus) and click inside the blue frame (approach go) to collect it, or not emit a response to not collect (approach no-go). Probabilistic outcomes (±20 cents) were presented immediately after go actions, or after a timeout period of 1.5 s had elapsed to define a no-go action. (*b*) Pavlovian conditioning. Subjects passively viewed fractal stimuli and heard auditory tones, deterministically followed after 1 s by wins and losses of 100, 10, 0, −10 or −100 cents for the best (henceforth labelled as ++), good (+), neutral (0), bad (–) and worst (– –) audiovisual Pavlovian conditioned stimuli (CSs), respectively. Tone frequency increased or decreased with CS value (counterbalanced). (*c*) Approach PIT stage. Subjects responded to mushrooms (instrumental stimuli) as before but now with fractals (Pavlovian CSs) tiling the background of the display and a tone corresponding to the fractal playing. No outcome was presented, but subjects were instructed to continue performing the instrumental task and that their choices counted towards the final total. No explicit instruction about the contribution of Pavlovian stimuli towards the final total was given. (*d*) To measure the acquisition of Pavlovian associations, passive Pavlovian conditioning trials (*c*) were interspersed with free choice trials administered on every fifth trial throughout Pavlovian conditioning (*d*). Here, subjects chose between two fractals presented concurrently. No outcome was presented, but subjects were told that the choices on these trials counted, with wins or losses added to the total provided at the end of the experiment. (*e*) Instrumental withdrawal training. As in approach training, except now subjects were told they were at home and had to throw away or not throw away mushrooms from their basket. They moved the cursor away from the mushroom and clicked in the empty blue frame to throw it away (withdrawal go) or did nothing to keep it (withdrawal no-go). (*f*) Withdrawal PIT. As in the approach PIT stage, the fractal stimuli tiled the background and subjects continued to perform the instrumental withdrawal task in extinction. For further details, see online Supplement S1. See online for the colour version of the figure.

The task consisted of two blocks, each in three parts (Fig. 1 and online Supplement S1). One block was an instrumental approach block where subjects first learned whether or not to collect mushrooms. Specifically, in this instrumental training, they were presented with six mushrooms and rewarded or punished for either collecting three mushrooms or not collecting the other three by approaching or not approaching them (Fig. 1*a*). Next, Pavlovian compound conditioned stimuli (CSs) were trained (Fig. 1*b*). In the PIT component itself, subjects were told to continue to choose either to collect or leave the mushrooms, but now the background was tiled with task-irrelevant Pavlovian CSs and outcomes were no longer explicitly presented (Fig. 1*c*). The PIT effect in the approach block is a bias in instrumental performance due to the presence of the Pavlovian CS. Even though the Pavlovian CS was actually uninformative about whether the mushroom should be collected or not, control subjects approached (collected) more mushrooms when the Pavlovian CS was positively valued and fewer when the Pavlovian CS was negatively valued (Huys *et al.* 2011).

Exactly the opposite was observed in the withdrawal block (Huys *et al.* 2011), which had the same sequence of three parts, except that subjects were asked to decide whether or not to throw away a mushroom by withdrawing from it, or keeping it by doing nothing (Fig. 1*e* and *f*). While in the approach block the active response involved movement towards the mushroom, in the withdrawal block it involved movement away from the mushroom. It was otherwise exactly the same. We previously found that, during the PIT part of the withdrawal block, appetitive CSs reduced the tendency to throw away mushrooms, while aversive CSs increased it (Huys *et al.* 2011). Hence the PIT effect was action-specific: when the active behaviour being modulated was approach, appetitive CSs promoted and aversive CSs inhibited it, while the opposite was true when withdrawal was being modulated.

We hypothesized that depression would reduce both appetitive and aversive PIT during approach. In addition, we examined whether reflexive Pavlovian processes might afford a less specific and more general guidance of instrumental choices by incidental valued stimuli by also testing whether action specificity is reduced. Finally, because we expected behaviourally observable PIT to parallel the internal biases of cognition by emotion, we predicted that these variables would be related to the longitudinal course of the disorder.

We recruited healthy controls and subjects with major depressive disorder (MDD) or dysthymia (DTH) in a naturalistic longitudinal follow-up study. In an attempt to examine the specificity of the findings with respect to depression, we also attempted to recruit patients with co-morbid anxiety (generalized anxiety disorder; GAD) and depression (MDD/DTH), and with anxiety (GAD) alone. All patients were re-contacted after 4 months to assess the state dependence of any effects that distinguished patients and controls at initial contact, as well as to examine whether these effects were related to future symptom course. As insufficient anxiety patients could be recruited, this report focuses on the depression dataset.

## Method

### Subjects and procedure

Patients were recruited from the Berlin area while in-patients at the Charité Hospital, via their community psychiatrists, or via self-referral through advertisements. Controls were recruited via advertisements and email alerts.

Prior to the experimental session, subjects completed the Structured Clinical Interview for DSM-IV-TR Axis I Disorders, Research Version, Patient Edition (SCID-I/P; First *et al.* 2002*b*) screening questionnaire, and the sections on mood disorder and GAD. Inclusion criteria were age 18–65 years and satisfying criteria for a current MDD or DTH or GAD. After the experimental session, all subjects underwent a full structured diagnostic interview performed by trained raters (First *et al.* 2002*a*, *b*). Self- and observer-rated scales (Hamilton, 1960; Beck *et al.* 1996*a*, 1988) and intelligence quotient (IQ) (German vocabulary test *Wortschatztest*; Schmidt & Metzler, 1992) were obtained at this point. For patients, exclusion criteria included Axis I disorders other than GAD, MDD or DTH, and for controls any current or past Axis I diagnosis. Additional exclusion criteria for all subjects were neurological, endocrine and cardiac disorder or use of drugs of abuse in the past 6 months. Patients were re-contacted after 4 months, and retested at T2 between 4 and 6 months after initial testing at T1. All testing sessions including follow-up were performed by E.F. and M.G. between February 2009 and November 2010 (inter-rater reliability on 10 patients assessed by both: *κ* = 0.92). All participants gave written informed consent and ethical approval was obtained from the ethics committee of Charité – Universitätsmedizin Berlin.

**Fig. 2.**
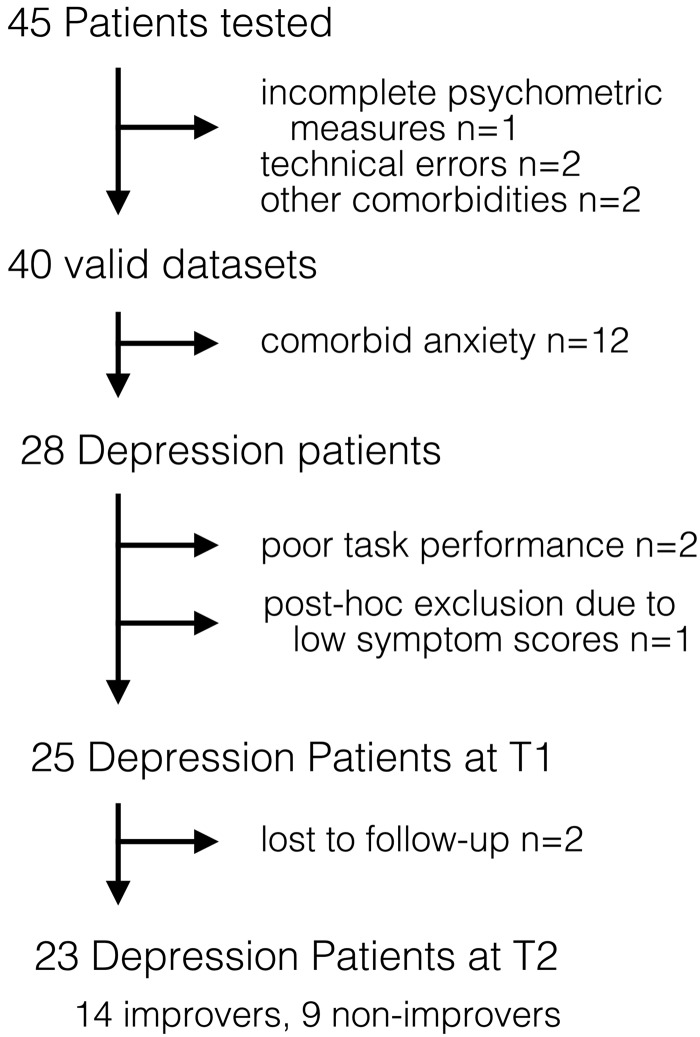
CONSORT (Consolidated Standards of Reporting Trials) diagram for patients in the study. A total of 45 patients with major depressive disorder, dysthymia and/or generalized anxiety disorder were recruited. Data were lost due to technical errors (*n* = 2), incomplete psychometric measures (*n* = 1) and due to co-morbidities identified in the Structured Clinical Interview for DSM-IV-TR Axis I Disorders (SCID) after task completion (*n* = 2), resulting in 40 valid datasets. Analyses focused on patients with depression only.

A total of 45 patients were tested on the task (Fig. 2). Analyses focused on the 28 patients without anxiety (MDD *n* = 26 or DTH *n* = 2) because insufficient patients with pure GAD (*n* = 1) or co-morbid GAD + depression (GAD + MDD *n* = 10 and GAD + DTH *n* = 1) could be recruited. Two MDD subjects were excluded due to performance-related issues (online Supplement S3) and one DTH subject *post-hoc* due to low symptom scores [Beck Depression Inventory (BDI) score <11 at T1]. Two of the patients were unavailable for follow-up, resulting in a final sample of 25 depressed patients at T1, 23 depressed patients at T2, and 40 healthy controls. Results including patients with co-morbid anxiety are included in the online Supplementary material. A total of 61 healthy controls were tested. Data for six controls were lost (three technical errors, three incomplete). Of the controls, 46 were part of our previously published PIT study (Huys *et al.* 2011). To allow for matching, nine healthy males over 35 years had to be added. From the total pool of 55 controls we extracted the 40 that matched the 40 patients best for age, sex and education (online Supplement S2).

### Analysis

#### Pavlovian and instrumental training

The number of correct choices on the Pavlovian free choice trials (Fig. 1*c*) was compared with chance level (0.5) for each subject individually using a binomial test.

Acquisition of instrumental training was assessed by examining the overall number of correct responses and comparing this with chance (50%). To examine the rate of learning, we computed a learning curve for each stimulus separately, averaged these within each subject and fitted a linear function of time to this learning curve. Tests were performed as in the Statistical tests section.

#### PIT behaviour

We computed the proportion of go responses in the presence of each of the five valenced Pavlovian CSs in the approach and withdrawal blocks separately. This analysis averaged over all instrumental stimuli within a block and was orthogonal to the relative value of go and no-go instrumental stimuli. Appetitive approach PIT was measured as the linear regression coefficient of the go probability across all instrumental stimuli for neutral and positive (0, +, ++) CSs. For aversive approach PIT, we regressed the go probability across all instrumental stimuli on neutral and negative (0, –,– –) CS values. For each action frame (approach and withdrawal), we then performed a single linear regression over all five CS values. Action specificity was calculated as the difference between the linear regression coefficients in approach and withdrawal blocks.

Planned analyses involved comparisons between controls and patients at T1, and between patients at T1 and T2 on action specificity, and on approach appetitive and approach aversive PIT.

#### Longitudinal course

The relationship of behavioural measures to longitudinal course was assessed by relating T1 behavioural measures to T2 depression scores. To control for baseline scores, we also regressed T2 scores onto T1 scores and asked whether behavioural measures were related to the residuals.

For categorical analyses, improvers were patients with a MDD or DTH diagnosis at T1 who achieved either a reduction in BDI scores greater than the median (a reduction of nine BDI points or more), or whose T2 BDI score was <50% of their T1 BDI score.

#### Statistical tests

Analyses were performed in Matlab. Outlier data points, i.e. data points >3 standard deviations from the relevant mean, were removed prior to performing any test. If there was a significant departure from Gaussianity (*p* < 0.05, Kolmogorov–Smirnoff test), we performed non-parametric tests (Wilcoxon signed-rank tests to test against null effects and Mann–Whitney *U* tests to compare populations) and otherwise *t* tests. For the effects of time we computed the statistics in a paired manner. Residual BDI scores at time T2 were computed by retaining the residuals after linearly regressing BDI T2 onto BDI T1 scores.

## Results

Table 1 shows subject characteristics. Controls and patients were matched for age, sex, IQ and education (online Supplement S2). Roughly half the patients were medication-free. Patients were, on average, moderately depressed at T1 and only mildly depressed at T2 (Beck *et al.* 1996*b*). Patients and controls acquired instrumental and Pavlovian contingencies equally and well at T1 and patients learned the instrumental task on retesting at T2 faster than at T1 (online Supplement S3).

**Table 1.** Subject characteristics

	Controls	Patients T1	Patients T2
*n*	40	25	23
Male, %	38	32	30
Age, years	27.3 (7)	28.3 (8)	
Education, years	12.8 (1.7)	13.3 (1.6)	
IQ	114.2 (10.1)	117.3 (10.6)	117.8 (10.6)
BDI-II	2.8 (3.7)	23.4 (8.6)*	14.5 (10.7)†
HAM-D	0.8 (1.4)	17.3 (5.6)*	9.1 (7.0)†
BAI	4.3 (3.8)	13.5 (7.8)*	9.7 (7.4)‡
HAM-A	0.6 (1.2)	14.8 (5.1)*	7.6 (6.8)†
Medication status, *n*			
None	40	13	13
SSRI	0	3	2
5-HT	0	3	3
Other	0	6	5

Data are given as mean (standard deviation) unless otherwise indicated.

IQ, Intelligence quotient; BDI, Beck Depression Inventory-II; HAM-D, Hamilton Depression Rating Scale; BAI, Beck Anxiety Index; HAM-A, Hamilton Anxiety Rating Scale; SSRI, only selective serotonin inhibitor; 5-HT, on one mainly serotonergic medication; other, lithium, antipsychotic medication, benzodiazepines or combination of multiple treatments.

* Significantly (*p* < 0.05) different from controls.

† Significantly (*p* < 0.05) different from T1.

‡ Trend difference from T1 (*p* < 0.1). All other comparisons are non-significant (*p* > 0.2).

### Appetitive and aversive PIT during approach

We first examined valence-specific PIT effects during approach only. We regressed each subject's response probability, averaged across all instrumental stimuli, onto neutral and positive (0, +, ++) CSs to measure appetitive PIT, or onto neutral and negative (0, –, – –) CSs to measure aversive PIT. Comparison of the resulting linear regression coefficients yielded no group differences in appetitive or aversive approach PIT at T1, nor was there an effect of time in patients (all *p* > 0.1).

### Action-specificity at T1

A central finding in one of our previous studies (Huys *et al.* 2011) was that the effects of Pavlovian CSs depended on the nature of the instrumental action, with positive *v.* negative CS valence promoting active approach *v.* active withdrawal, respectively. We computed approach and withdrawal PIT effects by regressing each subject's response probability during each of the two blocks on all five CS values (again averaging across instrumental stimuli). PIT action-specificity was the difference between the linear regression coefficients during approach and withdrawal.

**Fig. 3.**
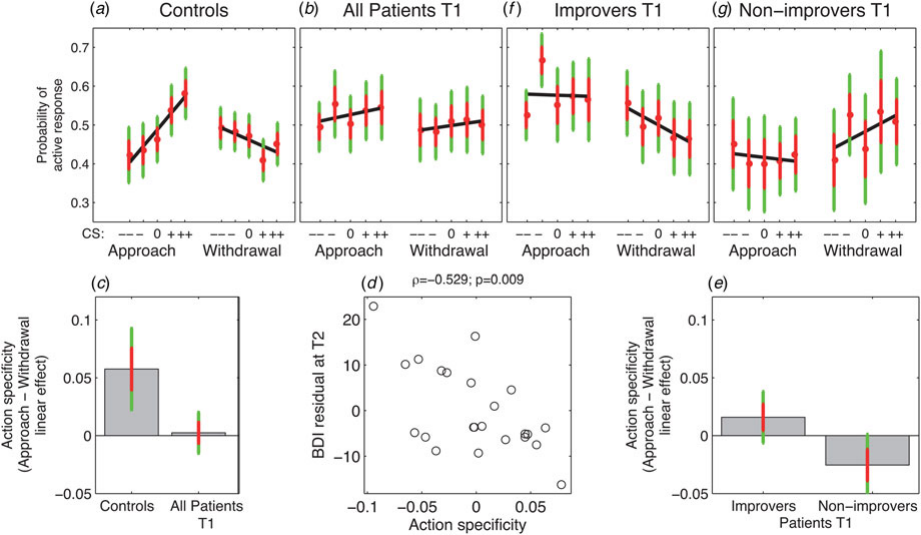
Pavlovian-instrumental transfer (PIT) data. (*a*) Choice data for control subjects show an action specificity, i.e. the valence of Pavlovian conditioned stimuli (CSs) is positively related to an active response during approach, but negatively during withdrawal (*p* = 0.002). (*b*) In major depressive disorder patients, PIT effects during approach and withdrawal did not differ, i.e. PIT effects are not action-specific (*p* = 0.7). (*c*) Action specificity (difference in linear CS valence effects between approach and withdrawal conditions) is trend-wise greater in controls than patients at T1 (*p* = 0.07). (*d*) The strength of action specificity correlates negatively with residual Beck Depression Inventory (BDI) score at follow-up T2, i.e. after correcting for BDI score at T1 (ϱ = −0.53, *p* = 0.009). (*e*) Action specificity is greater in those patients who go on to improve at follow-up compared with those who do not (*p* = 0.04). (*f*) and (*g*) PIT effects at T1 for improvers (*f*) and non-improvers (*g*). In panels (*a*), (*b*), (*f*) and (*g*), red dots show means, red error bars 1 standard error, green error bars 95% confidence intervals, and black lines are linear regressions (see online for the colour version of the figure).

At T1, PIT was action-specific in controls (*p* = 0.002, signed rank, Fig. 3*a*), but not in patients (*p* = 0.72, signed rank, Fig. 3*b*) and there was a trend-wise difference between patients and controls (*p* = 0.07, *U* test, Fig. 3*c*). Exploring approach and withdrawal blocks separately, there was a trend-wise difference between patients and controls during approach (*p* = 0.07, *U* test), but none during withdrawal (*p* = 0.8, *U* test), suggesting that any group difference in action specificity was driven more by approach than withdrawal effects at T1.

### Action-specificity at T1 predicts the course of depression

Some patients improved more than others over the follow-up period. The size of the improvement was proportional to action-specificity at T1: action-specific PIT scores correlated with the BDI scores at T2 (ϱ = −0.47, *p* = 0.02, Spearman rank correlation) and with the residual BDI scores after correcting for (regressing out) the BDI scores at T1 (ϱ = −0.53, *p* = 0.009, Spearman rank correlation, Fig. 3*d*).

Improvers (*n* = 14) and non-improvers (*n* = 9) had similar numbers of previous episodes of depression and hospitalizations and did not differ in depression severity at T1, but did so at time T2 (Table 2). While improvers showed PIT action-specificity at T1, non-improvers did not (group difference: *p* = 0.04, *U* test, Fig. 3*e*–*g*). Hence, patients who had intact action-specificity had a better outcome over the course of follow-up.

**Table 2.** Characteristics of improvers and non-improvers

	Improvers	Non-improvers	Statistics
Previous episodes	2.5 (3.1)	1.3 (1.3)	*p* = 0.7, *U* test
Hospitalizations	1.1 (0.7)	0.3 (0.5)	*p* = 0.6, *U* test
BDI T1	23.3 (9.8)	22.7 (5.8)	*p* = 0.9, *t*_21_ = 0.17
BDI T2	8.3 (6.2)	24.2 (8.8)	*p* = 4.6 × 10^−5^, *t*_21_ = −5.1

Data are given as mean (standard deviation).

BDI, Beck Depression Inventory.

Exploring these effects further, we found that the difference in PIT action-specificity between improvers and non-improvers was driven by withdrawal effects. At T1, withdrawal was modulated by Pavlovian stimuli in improvers but not so in non-improvers (*p* = 0.02 and *p* = 0.2, respectively, both signed rank tests), and the groups differed in the withdrawal but not the approach condition (*p* = 0.02 and *p* = 0.5, respectively, *U* tests). Furthermore, withdrawal PIT correlated with residual BDI scores at T2 (ϱ = 0.55, *p* = 0.01, rank correlation), while approach PIT did not (*p* = 0.2). Finally, action-specificity correlated with improvement across symptom subdomains, but most strongly with changes in anhedonic symptoms (online Supplement S4).

### Effects of medication, time and anxiety

There were no effects of medication, and the findings persisted when controlling for medication (online Supplement S5). In the depressed group, a trend for action specificity was present at T2 (*p* = 0.08, signed rank) but it did not interact with time and did not differentiate improvers from non-improvers (online Supplement S6).

All results held when including patients with co-morbid anxiety (online Supplement S7). At T1, there was no action specificity in patients *p* = 0.7. At T1, there was a significant difference between patients and controls (*p* = 0.04) and between improvers and non-improvers (*p* = 0.009). Action specificity at T1 also correlated negatively with residual BDI scores at T2 after correcting for scores at T1 (ϱ = −0.48, *p* = 0.005). Notably, there was a significant effect of time on withdrawal PIT when including GAD subjects (*p* = 0.04; online Supplementary Table S3).

## Discussion

Advances in understanding decision-making have identified multiple separate and parallel, but interacting decision-making systems (Killcross & Coutureau, 2003; Daw *et al.* 2005; Huys *et al.* 2012; Dolan & Dayan, 2013; Lee *et al.* 2014). PIT is one paradigmatic examination of how incidental valued stimuli interact with and influence ongoing decisions about how to act. In the context of depression, this may tap into how unrelated but affectively salient events are coupled to current thought and behavioural selection processes.

Pavlovian stimuli exerted action-specific effects in healthy subjects. During depressive episodes, this was absent, but the relative preservation of action specificity predicted a better recovery. Thus, this first in-depth PIT examination suggests that in the depressed state the guidance of choices afforded by Pavlovian stimuli is reduced and lacks specificity. The finer differentiation between different types of behaviours seen in controls is lost during a clinical episode of depression.

Two facets of depression appeared to be differentially associated with the Pavlovian modulation of approach and withdrawal, which jointly make up action specificity. The distinction between improvers and non-improvers was driven by differences during withdrawal. Thus a more advantageous disease trajectory characterized those patients with relatively more intact Pavlovian guidance of withdrawal behaviours. Conversely, a distinction between patients and controls was more prominent during approach. A tempting, though emphatically tentative, interpretation is that the blunted modulation of approach might contribute more to a trait distinction between patients and controls. This contrasts with an alteration in the modulation of avoidance relating more to the trajectory and repair of depression itself.

The (marginally) blunted influence of both appetitive and aversive CSs on approach was broadly in keeping with reports of a symmetric blunting in responses to affective material (Bylsma *et al.* 2008) in depression. However, a straightforward insensitivity to rewards and losses should also have expressed itself in impairments in the acquisition of the instrumental or Pavlovian tasks, which was not the case. Hence, to the extent that a reduced PIT effect captures a similar underlying process as blunting, this might relate more to aspects of the expression than to the acquisition of value.

The fate of negative emotional reactions, i.e. the modulation of aversive behaviours in depression, is subject to much debate. Some emphasize a likely increase (Beck *et al.* 1979; Roiser *et al.* 2012) such that patients suffering from mood and anxiety disorders show more reactive aggression against themselves and others (Monahan *et al.* 2001). Experience sampling studies have shown that aversive events have greater affective consequences in never-depressed monozygotic twins with a depressed co-twin (Wichers *et al.* 2007). However, a meta-analysis showed that emotional reactions to negative stimuli are, overall, blunted in MDD (Bylsma *et al.* 2008). Akin to the finding that appetitive rather than aversive stimuli potentiate startle reflexes in MDD (Allen *et al.* 1999), our data suggest more subtle effects than a simple increase or decrease of PIT, specifically an alteration in the balance between approach and withdrawal. Our data also suggest that the affective control of aversive behaviours (by both appetitive and aversive expectations) is of particular importance to recovery. This hints that aversive influences, such as inappropriate inhibition of withdrawal, may have an unduly large effect on the maintenance of depression, for instance, by inhibiting rather than promoting withdrawal in dangerous situations. Maladaptive avoidance may in turn facilitate individuals’ self-selection into high-risk environments (Kendler *et al.* 1999) and thereby set up vicious depression–stress cycles.

At a neural level, we have shown that behavioural suppression due to aversive CSs is related to both serotonin (Geurts *et al.* 2013*b*) and to how aversive CSs modulate connectivity between the ventromedial prefrontal cortex (extending into the subgenual cortex, itself also predictive of the course of depressive episodes: Mayberg, 2009; Fu *et al.* 2013) and the caudate nucleus (Geurts *et al.* 2013*a*), raising the possibility that a lack of action specificity during depression might be related to neurobiological deficits previously described in MDD (Mayberg *et al.* 2005).

The generalization to severely depressed clinical samples requires verification as the present study contained a large fraction of moderately depressed students with relatively high average IQ. Second, we did not find significant medication effects, though the study was neither designed nor powered to detect these, and due to the small number of non-improvers we were unable to differentiate between subjects whose condition deteriorated *v.* remained stable. Third, we did not assess depressive episode length. Modulation of withdrawal responses at T1 was predictive of better recovery over the observation period. Hence it was surprising to see significant modulation of withdrawal even in non-improvers at T2. One possibility is that the recovery of withdrawal modulation is an early sign of improvement appearing before symptomatic improvement, and might predict recovery over a longer follow-up period even in the non-improvers. Conversely, improvers may have been at this advanced stage already when they were tested at T1. Finally, it should be noted that the number of subjects is relatively small for a longitudinal study, limiting both robustness and power, and hence the ability to detect reliable predictive relationships.

In conclusion, the current results provide a strong motivation to pursue computationally inspired decision-making tasks to examine the structure of emotional processes in depression. Aspects of decision-making that have predictive value may become useful for the guidance of treatment or for alternative (and complementary) classifications of psychiatric disorders and individual patients.

## Supplementary material

For supplementary material accompanying this paper visit http://dx.doi.org/10.1017/S0033291715002597. click here to view supplementary material
