# Supplementary material for: The specificity of Pavlovian regulation is associated with recovery from depression
Source: Psychol Med. 2016 Apr;46(5):1027–35. doi: 10.1017/S0033291715002597 (PMC4825095; doi:10.1017/S0033291715002597)
Supplement: Supplementary file 1 [file S0033291715002597sup001.pdf]

SUPPLEMENTARY MATERIAL

S1 DETAILED TASK DESCRIPTION

INSTRUMENTAL TRAINING

By design, the instrumental task was difficult, with few instrumental stimulus repetitions, interleaved presentation and highly noisy reinforcement signals. Weakly reinforcing the instrumental tendency allowed for strong Pavlovian CS effects in the PIT part of the task (below). Specifically, the instrumental task was framed in terms of collecting and sorting mushrooms. There were two counterbalanced blocks: approach and withdrawal (Figure 1A,E). Each block contained six mushrooms. They were randomly selected from a pool of twelve and each presented 10 times, resulting in two blocks of 60 training trials. Subjects received reinforcement after every choice ("+20 cents" or "-20 cents") with probabilities of 0.75 or 0.25 depending on the stimulus. For half the mushrooms (three per block), a go response was rewarded with probability 0.75 and a nogo response with probability 0.25; for the other half, this reward contingency was reversed. Thus, each of the four types of action (approach go and nogo; and withdrawal go and nogo) were favoured by a greater chance of reward than punishment for three stimuli. Withdrawal was thus defined purely in terms of the intrinsically negative relationship of the action to the stimulus (moving away from it), and not in terms of any associated aversive outcomes (Roelofs *et al.*, 2009). The design therefore controls for any possible associations of activation or approach/withdrawal with predictions of reward versus punishment.

Thirty-one of the control subjects came from the pool of subjects reported in (Huys *et al.*, 2011), where the impact of deterministic and probabilistic feedback, and the impact of different types of withdrawal actions were tested. Overall, 35 controls experienced probabilistic feedback, as was the case for patients, and 5 deterministic feedback. Twenty-four used the throwaway action like patients. Sixteen performed the experiment with a different withdrawal action, which involved releasing the mouse button rather than moving the mouse cursor away from the mushroom. In the original experiment, detailed analyses revealed no difference between the two sorts of withdrawal action, or between deterministic and probabilistic instrumental feedback. We aggregated all the data. We note that the differences in instrumental reinforcement could, at worst, influence the overall strength of PIT effects, but it could not affect any differential influences of appetitive vs aversive CSs. Likewise, the variations in withdrawal actions could, at worst, influence the difference between approach and withdrawal, but could only do so in the controls. Neither of these variations can influence the pattern seen within the patients, or the association of depressive symptoms with the behaviour seen within the patient group.

PAVLOVIAN TRAINING

Five compound Pavlovian conditioned stimuli (CSs), consisting of a fractal visual stimulus and a pure tone were presented 12 times each in the first block, and 6 times in the second block (Figure 1B). Each stimulus was deterministically followed by one particular outcome (-1€, -0.1€, 0, +0.1€ or +1€). Every fifth trial was a forced choice trial: subjects were asked to choose between two Pavlovian stimuli in nominal extinction (no feedback; Figure 1D).

PAVLOVIAN-INSTRUMENTAL TRANSFER

In the third part of each block, the instrumental task was presented whilst the Pavlovian stimuli tiled the background of the display (Figure 1C,F). Subjects were told to continue with the instrumental task, again in nominal extinction. It is critical to note that the Pavlovian stimulus was presented over the entire background, and, as such, could not by itself modulate the directionality of actions.

FURTHER EXPERIMENTS

Further experiments on helplessness, including a task (Huys *et al.*, 2009) and questionnaires, will be reported elsewhere once analysed.

Follow-up

At follow-up, patients performed the task with a different, counterbalanced, stimulus set.

S2 MATCHING

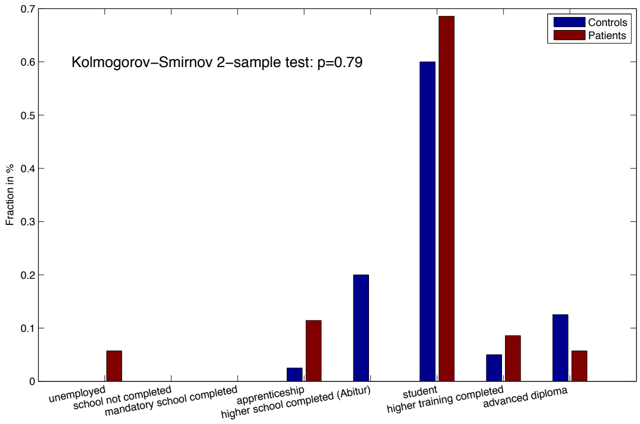

FIGURE S1: **Distribution of educational status between patients and controls.** A two-sample Kolmogorov-Smirnov test identified no significant difference ($p = 0.94$). 60% of the controls and 73% of the patients were currently students.

From the total pool of 55 subjects, we extracted the 40 that matched the sample of 40 patients best for age by first sorting patients in terms of average squared age difference to all controls. Starting with the patient with highest average age discrepancy, each patient was assigned the control subject with minimum age difference. This yielded a sample matched for age, sex and IQ (Figure S1).

S3 ACQUISITION OF PAVLOVIAN AND INSTRUMENTAL TRAINING

To test acquisition of Pavlovian conditioning, subjects intermittently chose amongst two Pavlovian stimuli in extinction (in the absence of feedback; Figure 1C). Two MDD patients performed no better than chance (binomial test, $p < .05$) and were excluded, resulting in a final sample of 40 controls, 25 patients at T1 (24 MDD, 1 DTH) and 23 patients with follow-up data at T2 (22 MDD, 1 DTH). Table 1 displays the group characteristics of this final sample.

Participants acquired instrumental approach and withdrawal successfully. At T1, both patients and controls chose better responses more than chance ($67 \pm 9.6\%$ and $63 \pm 9.4\%$; $p = 4 \times 10^{-9}, t(24) = 8.99$ and $p = 3 \times 10^{-10}, t(39) = 8.41$ respectively; difference not significant $p = 0.17, t(63) = -1.39$).

Patients learned better at T2 ($72 \pm 8.6\%$ correct) than at T1. This difference was significant ($p = 0.04, t(22) = -2.18$) and the linear learning trends during T2 were faster than during T1 ($p = 0.055, t(23) = 2.02$). Including the difference in learning trends as a covariate in a multiple regression abolished the session effect ($p = 0.16$), suggesting subjects' better performance better at T2 was due to faster learning.

S4 ASSOCIATION OF ACTION SPECIFICITY WITH IMPROVEMENT IN SPECIFIC SYMPTOM GROUPS

To examine whether action specificity might predict improvement in particular symptom domains, we examined four different subscores of the BDI (Table S1). Action specificity was most strongly associated with improve-

Score	BDI items in score	linear correlation	p
Anhedonic score	4, 12, 15, 21	-0.59	0.004
Somatic score	11, 16-18, 20	-0.51	0.01
Affective score	1-3, 4-10, 14	-0.51	0.01
Cognitive score	13, 19	-0.37	0.08

TABLE S1: Association of action-specificity at T1 with improvement in four subscores of the BDI. Pearson's linear correlation coefficients. p values not corrected for multiple comparisons.

ment in anhedonic symptoms. It was also associated with improvement in somatic, affective and (at trend level) cognitive symptoms. The correlation with anhedonic score improvement was significantly larger than the correlation with cognitive score improvement ($p = 0.03$).

S5 MEDICATION EFFECTS

To maximise power, we examined the effect of medication in the entire depression + GAD sample. First, we examined whether T1 action specificity depended on medication. A linear multiple regression analysis with the predictors group and medication showed a significant effect of group $p = 0.006$ but not of medication ($p = 0.3$). Similarly, in a separate analysis, the significant effect of the factor improver/nonimprover remained ($p = 0.01$) when including the medication factor; and this itself did not reach significance ($p = 0.08$), suggesting that medication did not explain the relationship between action specificity and improvement. Examining different types of medications (SSRIs, non-SSRI serotonergic medications or other medications (Lithium, mood stabilisiers, antipsychotics)) also failed to yield significant medication effects (all $p > 0.1$) and did not abolish the significant group effect at T1. Finally, we examined whether medication might account for the relationship between action specificity and BDI score at T2 by also regressing out medication status. The correlation between action specificity and the resulting residual BDI scores at T2 was still significantly negative $\rho = -0.3924$, $p = 0.03$.

S6 ACTION-SPECIFICITY: CHANGES OVER TIME

Action specificity was present in patients at T2 at a trend level ($p = 0.08$, signed rank). It no longer differentiated improvers from non-improvers ($p = 0.7$, signed rank; S2) and correlated neither with BDI scores at T2, nor with the change in BDI scores (both $p > .2$, rank correlation). Action specificity at T2 was driven by withdrawal PIT ($p = 0.003$, signed rank), with both improvers and non-improvers showing a significant effect at T2 ($p = 0.02$ and $p = 0.04$, respectively, signed rank tests). Effects of time on action specificity or withdrawal PIT did not reach significance in the depressed group (both $p > 0.1$).

We note that repetition effects may contribute to the behavioural findings at T2 in patients, and this needs to be ruled out using test-retest data in controls. However, that the ability to predict recovery is achieved based entirely on behavioural data at T1 only. Patients showed stronger instrumental behaviour at T2 and this may have negatively influenced our ability to see PIT effects at T2, but cannot by itself lead to false positives, particularly not at T1.

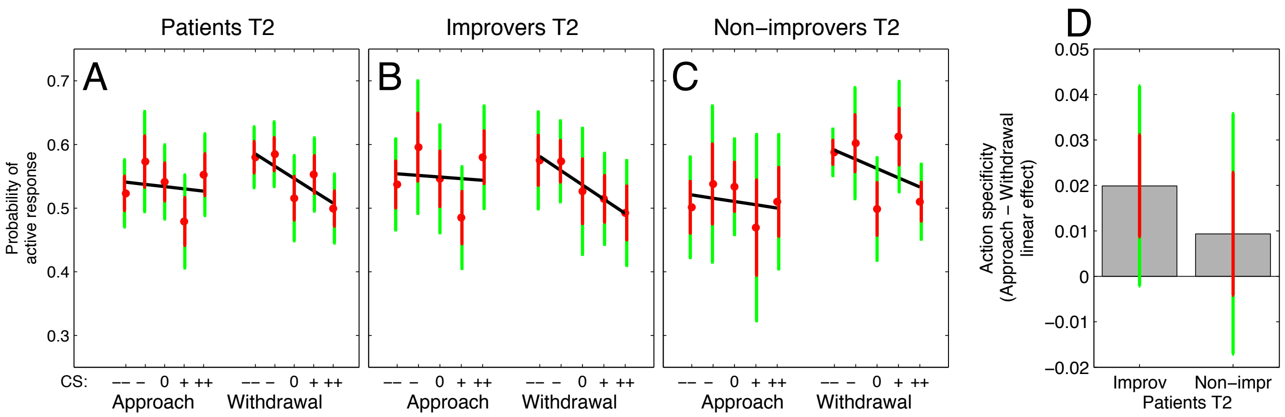

FIGURE S2: **PIT effects at T2. A**: Overall PIT effects of patients at T2. **B**: PIT effects of improvers at T2. **C**: PIT effects of non-improvers at T2. **D**: Action specificity of improvers and non-improvers at T2. Layout of A-C equivalent to that of Figure 3A-D.

S7 ANXIETY

TABLE S2: **Subject characteristics - depressed patients with + without GAD comorbidity.**

	Controls	Patients T1	Patients T2
Number	40	35	32
Male	38%	31%	31%
Age	27.3±7	28.4±8	
IQ	114.2±10.1	115.1±10.2	115.8±10.0
BDI	2.8±3.7	* 23.1±7.7	† 15.0±10.6
HamD	0.8±1.4	* 18.9±5.6	† 9.9±7.6
BAI	4.3±3.8	* 15.8±9.1	[1] 12.0±9.4
HamA	0.6±1.2	* 16.3±6.1	† 8.4±7.1
**Medication status**			
None	40	17	17
SSRI	0	5	4
5HT	0	5	5
Other	0	8	6

$*$ significantly ($p < 0.05$) different from controls. † significantly ($p < 0.05$) different from T1. [1] trend difference from T1 ($p < .1$). All other comparisons are non-significant ($p > 0.2$). BAI = Beck Anxiety Index; BDI = Beck Depression Index II; HamD = Hamilton Depression score; SSRI = only selective serotonin inhibitor; 5HT = on one mainly serotonergic medication; Other = Lithium, antipsychotic medication, benzodiazepines or combination of multiple treatments. One patient with comorbid DTH+GAD was excluded due to poor performance on the Pavlovian forced choice trials. One patient with MDD+GAD was unavailable for follow-up.

TABLE S3: **Results in depression only group (24 MDD, 1 DTH) are maintained when including patients with co-morbid GAD (24 MDD, 1DTH, 10 MDD+GAD).**

Measure	Contrast	Depression only		Comorbid	
Action specificity	T1 in patients	$p = 0.716$		$p = 0.688$	
	T2 in patients	$p = 0.078$		$p = 0.060$	
	T1 patients vs controls	$p = 0.073$		$p = 0.040$	
	T1 improvers vs nonimprovers	$p = 0.041$		$p = 0.009$	
	Correlation with residual BDI at T2	$p = 0.009$	$\rho = -0.529$	$p = 0.005$	$\rho = -0.483$
Withdrawal PIT effect	T1 improvers	$p = 0.017$		$p = 0.010$	
	T1 non-improvers	$p = 0.200$		$p = 0.420$	
	T1 improvers vs non-improvers	$p = 0.019$		$p = 0.012$	
	T2 improvers	$p = 0.020$		$p = 0.008$	
	T2 non-improvers	$p = 0.039$		$p = 0.003$	
	T2 improvers vs non-improvers	$p = 0.682$		$p = 0.012$	
	Time effect T1 x T2	$p = 0.108$		$p = 0.043$	

All tests are as described in the text, either Wilcoxon signed-rank or Mann-Whitney U tests. Results in column 'Depression only' are as reported in the main text. One patient with comorbid DTH+GAD was excluded due to poor performance on the Pavlovian forced choice trials. One patient with MDD+GAD was unavailable for follow-up.
